# Integration of single-cell transcriptome of female early gonadal development in bovine, goats, and pigs

**DOI:** 10.1016/j.isci.2026.116074

**Published:** 2026-05-22

**Authors:** Shiyao Han, Qianhui Zou, Yifei Fang, Junmei Zhang, Yue Su, Shengcan Xie, Zhen Yang, Yiyu Zhao, Ningxiao Li, Wenjing Wan, Linxiu Yue, Heshuangyi Xie, Yulei Wei, Ellie Duan, Young Tang

**Affiliations:** 1 Shaanxi Centre of Stem Cells Engineering & Technology, Key Laboratory of Livestock Biology, College of Veterinary Medicine, Northwest A&F University, Yangling 712100, China; 2 Guangdong Provincial Key Laboratory of Brain Connectome and Behavior, Brain Cognition and Brain Disease Institute (BCBDI)(Chan, 2021 #1), Shenzhen-Hong Kong Institute of Brain Science, Shenzhen Institutes of Advanced Technology (SIAT), Chinese Academy of Sciences (CAS), Shenzhen 518055, China; 3 Cornell University, Department of Animal Science, Ithaca, NY 14853, USA; 4 State Key Laboratory of Animal Biotech Breeding, College of Biological Sciences, China Agricultural University, Beijing, China

**Keywords:** developmental anatomy, animals, transcriptomics

## Abstract

The molecular programs governing early female gonadal development in livestock remain incompletely characterized. Here, we generated a single-cell transcriptomic atlas of bovine fetal ovaries spanning sex determination to early folliculogenesis and performed an integrated cross-species comparison with goats and pigs. We identified major germline and somatic cell populations and reconstructed germ cell differentiation trajectories, revealing conserved stages of pluripotency, retinoic acid (RA) response, and meiotic initiation, alongside species-specific developmental dynamics. Notably, goats exhibited delayed meiotic progression compared with bovine and pigs. Transcription factor analysis uncovered both conserved and bovine-specific regulators in granulosa cells. Cell-cell communication analysis further highlighted prominent RA-responsive MDK/PTN-NCL signaling and increased network interactions during meiotic entry in bovine and porcine but absent in pre-meiotic goat gonads. These findings provide a cross-species framework for early ovarian development in livestock and offer novel insights into mammalian gametogenesis and reproduction.

## Highlights

• A single-cell atlas of early bovine ovarian development
• Cross-species analysis reveals conserved and species-specific germ cell differentiation
• Goats exhibit delayed meiotic progression relative to cattle and pigs
• RA-responsive MDK/PTN-NCL signaling shapes the microenvironment for meiotic entry

## Graphical abstract

**Figure:**
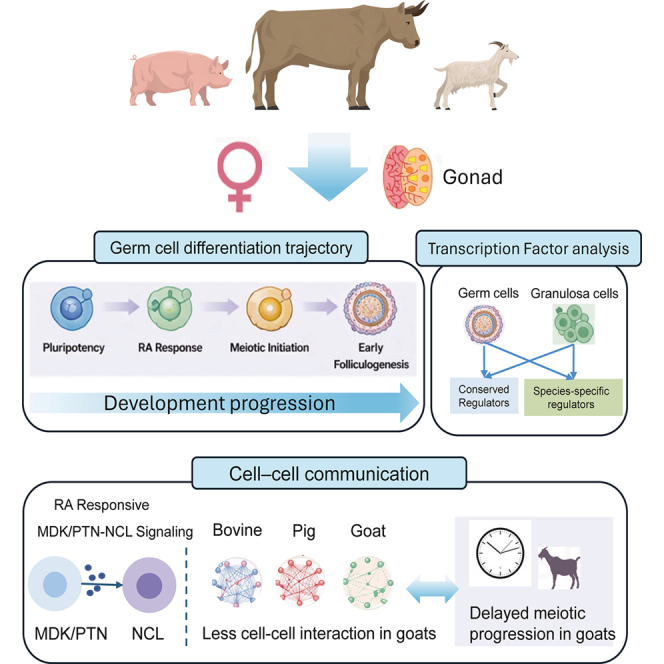

## Introduction

Sexual reproduction in mammals relies on the precise development of male and female gonads, a process initiated from the bipotential embryonic genital ridge.^1^ The fate of this primordium is determined by a complex cascade of genetic and cellular events, leading to the differentiation of somatic support cells (Sertoli in males and granulosa in females) and the establishment of a niche for germ cell development.^2^ In females, primordial germ cells (PGCs) enter meiosis shortly after the differentiation of granulosa cells and subsequently arrest at the diplotene stage.^3^ This process is cooperatively promoted by the activation of the *RSPO1/WNT4*/β-catenin pathway^4^^,^^5^^,^^6^^,^^7^^,^^8^^,^^9^ and *FOXL2*.^10^^,^^11^ In contrast, in male gonads, expression of *SRY* followed by *SOX9* and *CITED4* drives the differentiation of Sertoli cells,^12^^,^^13^^,^^14^ and germ cells become mitotically arrested after sex determination.^15^ While the core principles of sex determination are established, the complete regulatory landscape, particularly the intricate crosstalk between germ cells and somatic cells, remains an area of active investigation.

Our understanding of these processes is largely derived from studies in mice and humans. These models have revealed conserved regulators of PGC specification like *PRDM1*, *PRDM14*, and *TFAP2C*.^16^^,^^17^^,^^18^ However, critical species differences also exist. For example, *SOX2* is expressed in mouse but repressed in human PGCs, while *SOX17* is essential only for the specification of human PGCs.^19^^,^^20^ These species-specific regulations indicate that a truly comprehensive view of mammalian gametogenesis requires a broader phylogenetic perspective. Particularly, major livestock species, such as goats, pigs, and cattle, represent distinct evolutionary lineages and are vital for global agriculture. Despite their importance, the molecular choreography of early gonadal development in these species remains poorly characterized at single-cell resolution.^21^ While recent single-cell RNA sequencing (scRNA-seq) studies have provided valuable insights into the early gonadal development of goats and pigs,^22^ comprehensive comparative analyses across these species are lacking. Cattle, in particular, have been notably absent from such studies, despite their significant role in agriculture and unique reproductive characteristics. Moreover, a systematic, integrated cross-species comparison is lacking, leaving a gap in our knowledge of both conserved and species-specific early gonadal developmental programs. For instance, while some transcriptional regulators and signaling pathways, such as *FOXL2*^23^ and WNT/β-catenin, are conserved across species, the regulation of gonadal development can vary substantially between livestock species due to differences in reproduction strategies, gestational length, and embryonic development.^24^

Here, we present a single-cell transcriptomic atlas of fetal bovine ovarian development across key gestational stages and integrate these data with existing dataset from goats and pigs. This study aims to (1) define the cellular composition and transcriptional trajectories of germ and somatic cells during early female gonadogenesis; (2) identify conserved and species-specific transcriptional regulators; and (3) decipher the cell-cell communication networks that orchestrate these key developmental processes. Our work provides a foundational resource that not only elucidates the fundamental biology of gonadal development in ruminants and monogastric but also opens new avenues for advancing precision breeding and *in vitro* gametogenesis technologies.

## Results

### A single-cell atlas of early bovine ovarian development

**Figure 1:**
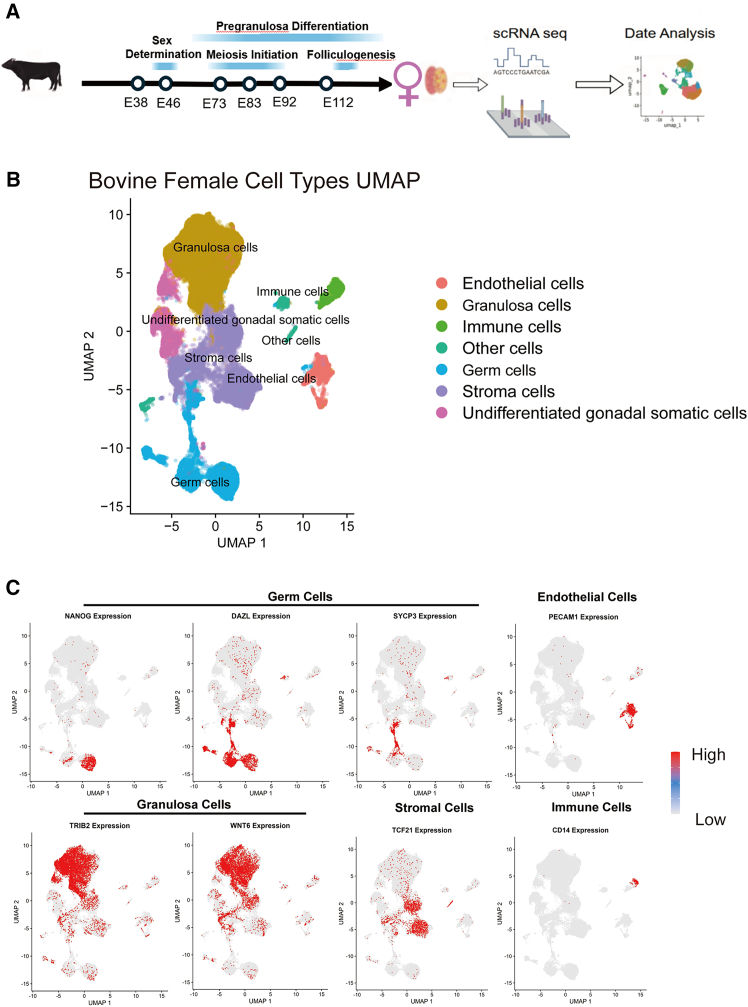
A single-cell atlas of early bovine ovarian development (A) Schematic diagram of experimental workflow for female bovine gonad analysis. (B) Uniform manifold approximation and projection (UMAP) visualization of female bovine gonadal germ cells and gonadal somatic cells. Cells are color-coded according to cell type. (C) UMAP embedding visualization of bovine germ cells and different gonadal somatic cells. Cells were color-coded based on normalized gene activity.

To define the cellular landscape of early female gonadal development in cattle, we performed scRNA-seq on fetal bovine ovaries across six gestational stages (E38, E46, E73, E83, E92, and E112) together with the data for an E50 female bovine gonad,^25^ these samples cover several key events including sex determination, pre-granulosa differentiation, meiosis initiation, and folliculogenesis (Figure 1A). By integrating the sequencing data from all seven stages, 54,268 high-quality cells were retained for analysis. Germ cells and gonadal somatic cells were first identified by automated marker-based annotation and then manually curated based on the enrichment of marker genes previously established in mouse^26^ and human^27^ studies, resulting in 8,221 germ cells and 46,047 somatic cells.

Unsupervised clustering identified seven major cell types (Figure 1B; Table S1). Germ cells are annotated by established markers, such as *POU5F1*, *NANOG*, and *DAZL* (Figures 1C and S1A). The somatic compartments consisted of granulosa cells expressing *TRIB2* and *WNT6*, stromal cells with *PDGFRA* and *TCF21*, endothelial cells expressing *PECAM1*, undifferentiated gonadal somatic cells with *LHX9* and *WT1*, and immune cells expressing *PTPRC* and *CD14* (Figures 1C and S1A). The “Other cells” refers to a cluster expressing kidney-related genes (OSR1, PAX2/8) most likely from cells of mesonephros tissue.

Our analysis reveals that bovine female PGC development shares considerable conservation with humans and mice, including core pluripotency factors for early PGCs (*POU5F1*, *NANOG*, and *NANOS3*), germ cell-specific markers such as *DDX4* and *DAZL*, meiotic markers *SYCP1*, *SYCP3*, and primary oocyte marker *FIGLA* (Figures 1C and S1A). Bovine granulosa cells exhibited significant enrichment of *TRIB2* (Figure 1C), which was known as a critical regulator for bovine ovarian folliculogenesis,^28^^,^^29^ and was also detected in porcine and goat granulosa cells.^22^^,^^30^ Another granulosa fate determinant, *RUNX1*,^31^ was also detected in bovine granulosa cells (Figure S1A). Similar to humans,^20^ *SOX17* is expressed in bovine germ cells (Figure S1A), indicating a conserved role for *SOX17* in bovine PGC development.

### Cross-species germ cell developmental trajectories reveal conserved programs and divergent meiotic timing

**Figure 2:**
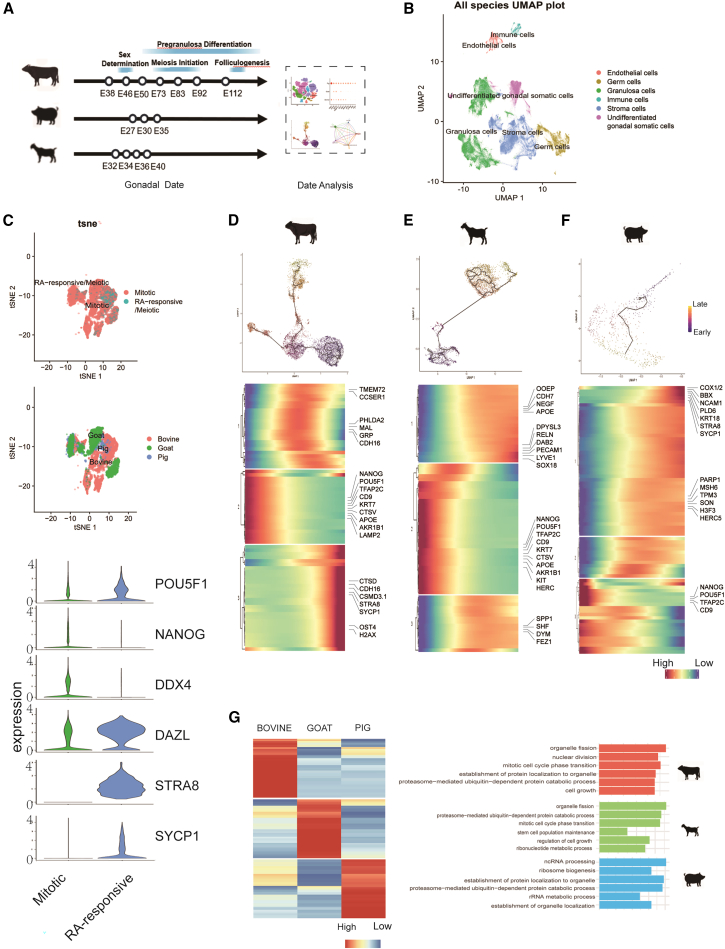
Cross-species germ cell trajectories reveal conserved programs and divergent meiotic timing (A) Schematic diagram of experimental work flow for female bovine, porcine, and goat gonad analysis. (B) t-stochastic neighborhood embedding (t-SNE) visualization of female gonadal cell clusters from bovine, pigs, and goats. Cells are color-coded according to different cell types. (C) Left: t-SNE visualization of female germ cells from bovine, pigs, and goats. Cells are color-coded according to different cell types and species. Right: the expression levels of representative markers for relevant cell types. (D–F) Top: pseudotime trajectories of female germ cells in bovine, pigs, and goats. Bottom: heatmap for gene expression levels along the pseudotime trajectories. Representative genes were listed (right). (G) Left: heatmap for DEGs identified among female bovine, porcine, and goat gonadal germ cells. Right: GO analysis based on these DEGs.

We next integrated our bovine data with public scRNA-seq datasets from female goat (E32, E34, E36, and E40) and porcine (E27, E30, and E35) gonads^22^ to conduct a cross-species comparative analysis (Figure 2A; Table S2). Integrated clustering of all three species robustly separated germ cells from somatic populations (Figure 2B). Germ cells were further separated into a mitotic PGC group, characterized by *POU5F1* and *NANOG*, and a retinoic acid (RA)-responsive/meiotic PGC group that expressed *STRA8* and *SYCP1*. Interestingly, both *DDX4* and *DAZL* were detected in both mitotic and RA-responsive/meiotic groups (Figure 2C).

To further define the dynamics of transcriptional regulation in female gonadal germ cell development, we reconstructed pseudotime trajectories for germ cells of each species (Figures 2D–2F). Early stages across all species were marked by high expression of pluripotency factors (*POU5F1*, *NANOG*, and *TFAP2C*), and the cell migration and homing receptor *CD9*^32^ (Figures 2D–2F). Bovine and goat early trajectories express cytoskeleton gene *KRT7*, lysosomal protease gene *CTSV*,^33^ lipid and steroidogenesis metabolism gene *APOE*,^34^ and *AKR1B1*—encoding the rate-limiting enzyme in polyol pathway^35^ (Figures 2D and 2E). In bovine and porcine early trajectories, the lysosome/autophagy-related gene *LAMP2*^36^ was highly expressed (Figures 2D and 2F), whereas in goat and porcine early trajectories, PGC marker *KIT* and ubiquitin ligase *HERC5* were highly expressed (Figures 2E and 2F). A key divergence was the timing of meiotic entry: *STRA8* and *SYCP1* were highly expressed in the middle-to-late stages of bovine and porcine trajectories but were not detected in goats (Figures 2E and 2F), indicating uninitiated meiosis up to the sampling stage (E40). This observation was consistent with the proposed onset of meiosis (∼E56) in female goat gonads.^37^^,^^38^

Various differentiation genes are expressed across early, middle, to late developmental stages in each species, such as *CTSD*,^39^ *CDH16*,^40^^,^^41^ and *CSMD3.1*^42^ in bovine (Figure 2D), *DPYSL3*,^43^ *RELN*, *DAB2*,^44^ *PECAM1*, *LYVE1*, and *SOX18*^45^ in goats (Figure 2E), and *COX1/2*,^46^^,^^47^ *BBX*,^48^ *NCAM1*, *PLD6*,^49^ *RDX*,^50^ and *KRT18* in pigs (Figure 2F). These data indicate that early female germ cell differentiation in livestock follows a broadly conserved trajectory including early PGC marker expression, lysosomal activity regulation, RA-responsive and meiosis onset, yet with species-specific dynamics for the expression of differentiation regulators.

To further investigate the conservation and divergence of germ cell development in livestock, we identified differentially expressed genes (DEGs) across female bovine, goat, and porcine germ cells (Figure 2G; Table S3). Gene Ontology (GO) analysis on upregulated DEGs revealed enrichment on biological process (BP) for “proteasome-mediated ubiquitin-dependent protein catabolic process” across all three species, while “organelle fission” and “mitotic cell cycle phase transition” were shared between bovine and goats, and “establishment of protein localization to organelle” was common to bovine and pigs (Figure 2G). These findings suggest that while DEGs vary by species, some of their regulatory roles in PGC development may be evolutionarily conserved. On the other hand, species-unique terms were identified, such as “stem cell population maintenance” and “ribonucleotide metabolic process” in goats; and “ribosome biogenesis”, “rRNA metabolic process”, and “establishment of organelle organization” in pigs, suggesting distinct regulatory emphases during PGC development.

### Cross-species comparative analysis of female gonadal somatic cells in livestock

**Figure 3:**
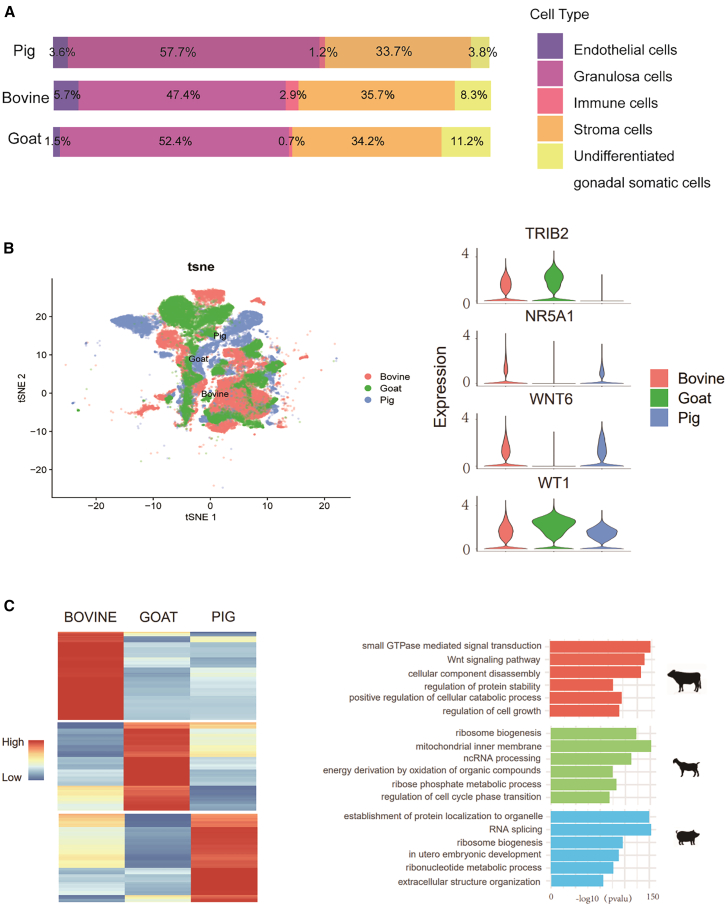
Cross-species comparative analysis of female gonadal somatic cells in livestock (A) Proportion of different types of female gonadal somatic cells in bovine, pigs, and goats. (B) Left: t-SNE visualization of granulosa cells from female bovine, goat, and porcine gonads. Cells are color coded according to different cell types and species. Right: the expression levels of representative markers for relevant cell types. (C) Left: heatmap for DEGs identified among female bovine, goat, and porcine gonadal granulosa cells. Right: GO analysis based on the DEGs.

Integrating female gonadal scRNA-seq data from bovine, goats, and pigs identified 5 gonadal somatic cell clusters: granulosa cells, stromal cells, endothelial cells, immune cells, and undifferentiated somatic cells (Figure 2A). The cellular architecture was conserved across species, with granulosa (47.4%–57.7%) and stromal cells (33.7%–35.7%) constituting the majority population (Figure 3A).

Expression of key granulosa cell markers varied significantly by species (Figures 3B and S1B). For example, *TRIB2* was highly expressed in bovine and goats, *WNT6* in bovine and pigs, and *AMHR2* was predominantly porcine. *NR5A1* expression was markedly weaker in goats, in line with its low expression compared to humans, macaques, and pigs^22^ (Figure 3B). Interestingly, *FOXL2*, a critical granulosa cell determinant in humans and mice,^27^ was only weakly detected in bovine and pigs and not detected in goats for the sampled stages (Figure S1B), in contrast to its robust expression in adult bovine follicular granulosa cells (Figure S1C). These results indicate species-specific and temporally regulated role of *FOXL2* for early ovarian development in livestock.

To assess functional conservation and divergence in granulosa cells development across the three species, we performed GO analysis on upregulated DEGs enriched in each species (Figure 3C; Table S4). In cattle, enrichments for “small GTPase-mediated signal transduction” and “Wnt signaling pathway” highlight the importance of these signals in granulosa cell development. In goat, “mitochondria inner membrane” and “energy derivation by oxidation of organic compounds” were enriched, implicating a unique role for energy metabolism in goat granulosa cells. In pigs, “*in utero* embryonic development” was enriched, suggesting a species-specific developmental regulation (Figure 3C). Overall, these results showed shared somatic cell composition with divergent, species-specific regulatory programs during granulosa cell differentiation in livestock species.

### Conserved and species-specific transcriptional networks govern germ and somatic cell fate

**Figure 4:**
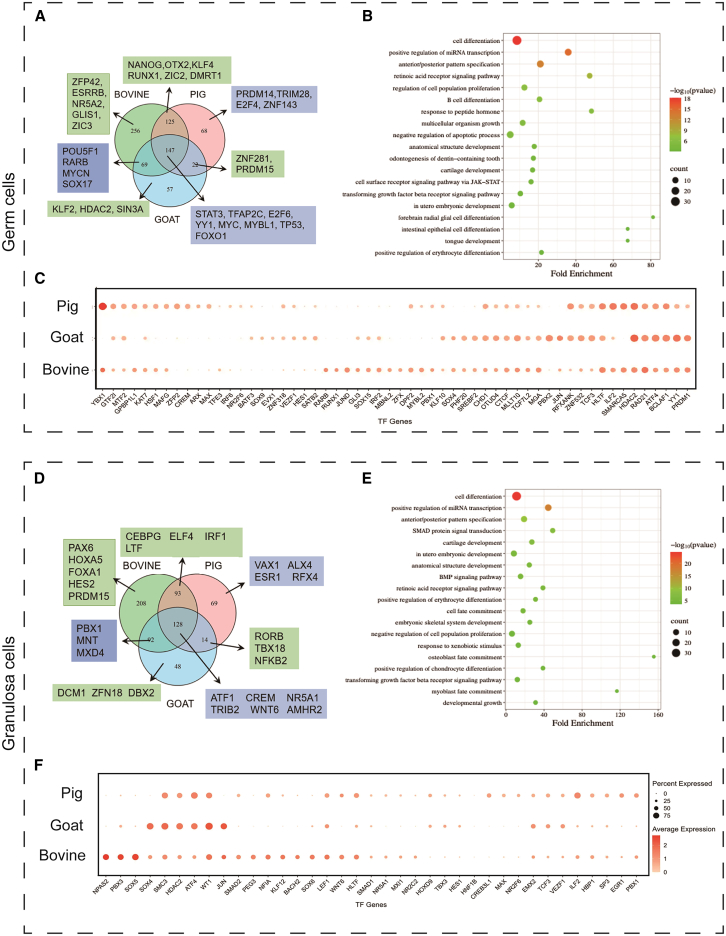
Conserved and species-specific transcriptional networks govern germ and somatic cell fate (A) Venn diagram showing the regulons overlapping in female germ cells of bovine, pigs, and goats. Key germ cell regulons are listed. (B) Top 20 GO terms based on conserved regulons among the three species. (C) Dot plot showing mean expression levels of representative TFs in female germ cells of bovine, pigs, and goats. The percentage of cells expressing each gene was indicated by dot size. Expression levels were color coded and derived from logarithm-scaled normalized counts. (D) Venn diagram showing the regulons overlapping in granulosa cells of female bovine, pigs, and goats. Key germ cell regulons are listed. (E) Top 20 GO terms based on conserved regulons among the three species. (F) Dot plot showing mean expression levels of representative TFs in granulosa cells of female bovine, pigs, and goats. The percentage of cells expressing each gene was indicated by dot size. Expression levels were color coded and derived from logarithm-scaled normalized counts.

To further define the transcriptional regulatory networks underlying gonad developmental programs, we performed transcription factor (TF) network analysis using pySCENIC (Table S6). In germ cells, 145 conserved TFs were identified across the three species (Figure 4A), including pluripotency regulators such as *STAT3* and *TFAP2C*,^51^^,^^52^ the cell cycle regulators such as *E2F6*, *E2F8*, *MYC*, *MYB*, *MYBL1*, *TP53*, and *FOXO1*,^53^^,^^54^^,^^55^^,^^56^ and the RA-responsive factors *RARA* and *RARG* (Figure 4A; Table S5). GO analysis for the conserved TFs identified enrichment terms for multi-organ, tissue, and cell type development, RA receptor signaling pathway, growth hormone/cell surface receptor signaling pathways via JAK-STAT3, and transforming growth factor beta (TGFβ) receptor signaling pathway (Figure 4B; Table S6). GO terms related to *in utero* embryonic development and mammary gland development were also enriched (Figure 4B; Table S6).

We next compared the expression of TFs across three livestock species. Several TFs shared high expression among all female germ cells were identified, including PGC regulator *PRDM1*, mitosis/meiosis regulators *RAD21*, *CHD1*, the stress-response and translational control factor *ATF4*,^57^^,^^58^ cell proliferation factor *YY1*, the epigenetic regulators *KAT7*, *MLL10*, the critical oocyte regulator *HDAC2*,^59^ the PRC2 component *MTF2*, the genome organizer *CTCF*, and the SWI/SNF component *HITF* (Figure 4C; Table S7). These common TF enrichments highlight the central role of chromatin remodeling in livestock PGC development.

Another SWI/SNF family member, *SMARCA5*, which is essential for meiotic progression in mouse germ cells,^60^^,^^61^ was expressed only in meiotic-progressing bovine and porcine germ cells but absent in pre-meiotic goats, consistent with the developmental timing (Figure 4C). In addition, the H3K4me2 reader *PHF20* was highly expressed in bovine and goats, whereas expression of chromatin organizer *SATB2* was higher in goats and pigs (Figure 4C). These data revealed conserved as well as species-specific epigenetic reprogramming regulators for female germ cell development in livestock.

Similarly, we performed pySCENIC analysis to identify TFs involved in granulosa cell development across species (Table S9). We identified 128 conserved TFs in granulosa cells, including gonadal somatic determinants, such as *GATA4*, *WT1*, *NR2F1*, *LHX9*, and *FOXO1*, the cell cycle and differentiation regulators, such as *FOS*, *FOSL1*, *ETS1*, RA-receptors *RARA*, *RARG*, *RXRG*, and the lipid metabolism regulator *PPARG* (Figure 4D; Table S8) GO enrichment analysis revealed pathways closely associated with gonadal development, including cell fate determination, BMP signaling, TGF-β receptor signaling, and RA receptor signaling (Figure 4E). Additionally, SMAD protein signal transduction and *in utero* embryonic development were also enriched, highlighting key regulatory networks involved in sex differentiation. (Figure 4E; Table S9).

We further compared the expression of the identified TFs in granulosa cells. Across the three species, highly expressed TFs included key genital ridge and gonadal somatic determinants *WT1* and *EMX2*,^62^^,^^63^^,^^64^ BMP signal mediator *SMAD1*, WNT signal mediators *LEF1* and *TCF3*, cell survival and stress-response gene *ATF4*, cell proliferation factor *SMC3*, *SP3*, the epigenetic regulators *HDAC2* and *HITF* (Figure 4F; Table S10). In contrast, *SMAD2*, *WNT6*, *MXI1*, and *PBX1* were expressed strongly in bovine and pigs, whereas *SOX4* and *JUN* were expressed more in bovine and goats, and *HOXD9*, *TBX3*, and *HES1* were expressed more in pigs and goats. Interestingly, *NPAS2*, *PBX3*, and *SOX5* were only highly expressed in bovine granulosa cells (Figure 4F). Among these bovine-specific TFs, *PBX3* is associated with primate granulosa cell development,^27^ and *SOX5* is expressed in mouse gonadal somatic cells and germ cells, with loss-of-function causing female-to-male reversal in Medaka fish.^65^ Together, these data suggest potential regulatory roles of these TFs in cattle ovary development.

### A retinoic acid-responsive signaling axis defines the meiotic microenvironment

**Figure 5:**
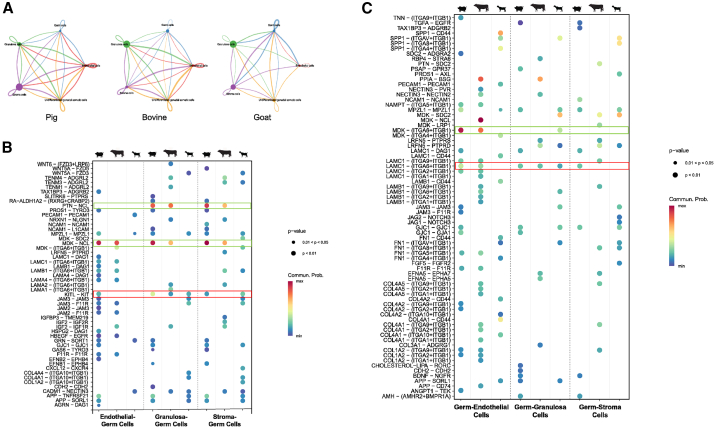
A retinoic acid-responsive signaling axis defines the meiotic microenvironment (A) Overall cell-cell communication network in bovine, goat, and pig gonadal tissues. (B) Dot plot depicting representative ligand-receptor interactions of gonadal endothelial, granulosa, and stromal cells to germ cells in female pigs, bovine, and goats. (C) Dot plot depicting representative ligand-receptor interactions of germ cells to endothelial, granulosa, and stromal cells in female pigs, bovine, and goats.

We used CellChat^66^ to map conserved and species-specific cell-cell communication networks in bovine, goats, and pigs (Figures S2A and S2B). Within each species, cell-cell communications appear stronger among the three somatic populations (stromal cells, endothelial cells, and immune cells) and also between germ cells and non-granulosa somatic cells than between granulosa and germ cells (Figure 5A). To identify pivotal ligand-receptor interacting pairs, we further compared the signals sent from three gonadal somatic populations to germ cells (Figure 5B), and from germ cells to each of the somatic cell population (Figure 5C). Across all species, two pairs of interactions were conserved: the canonical germ cell development *KITL-KIT*^67^ (Figure 5B) signaling from granulosa to germ cells and the extracellular matrix (ECM)-integrin signal *LAMC1*-(*ITGA6+ITGB1*) (Figure 5C) from germ cells to granulosa. Additionally, the *KITL-KIT* interaction was found to also initiate from endothelial cells to germ cells in pigs and from stromal cells to germ cells in pigs and goats (Figure 5B).

The ECM components, laminin and collagen, play critical roles in supporting spermatogenesis in testis, and reduction of *LAMA1* correlates with the loss of spermatogenesis in humans.^68^^,^^69^ We identified that between germ cells and granulosa cells, different species showed distinct ECM ligand and receptor pairs: in cattle, *LAMA1/2-(ITGA6+ITGB1)* and *COLA4A1-ADGRG1*; in goats, *LAMB1-(ITGA6+ITGB1)*, *COLA4A1/4-(ITGA10+ITGB1)*, and *FN1-(ITGAV+ITGB1)*; and in pigs, LAMC1-DAG1 (Figures 5B and 5C). These data indicate conserved as well as species-specific ECM signals for germ cell/granulosa communication, potentially regulating cell survival and differentiation for female livestock gonad development, similarly as they do in human male gonads.

Intriguingly, a robust Midikine-Nucleolin (*MDK-NCL*) interaction consistently presents in female bovine and pigs that is initiated from all three somatic cell populations to germ cells (Figure 5B). Pleiotrophin (*PTN*) also exhibited a strong *PTN-NCL* interaction that initiates from granulosa and stromal cells to germ cells in bovine and pigs (Figure 5B). *MDK* and *PTN* are retinoic acid (RA)-responsive heparin-binding growth factors abundantly expressed during mouse urogenital development^70^^,^^71^ and in human amniotic fluids.^72^ *MDK* was also identified in human granulosa cells, follicular fluid, and medulla of adult ovaries.^73^ In contrast, the *MDK-NCL* or PTN-NCL axis was absent in pre-meiotic goat gonads (Figure 5B). These data thus implicate a critical role of RA-responsive MDK/PTN-NCL signaling axis for the establishment of meiotic microenvironment in female gonads.

We further analyzed the interactions among gonadal somatic cells (granulosa, stromal, and endothelial cells) across species (Figures S3A–S3C). Although the overall numbers of germ cell-somatic interactions appear similar across the three species (Figures 5B and 5C), there are obviously more extensive inter-somatic cell communications in bovine and porcine gonads than in goats (Figures S3A and S3B), and no cell-cell interaction was identified from goat endothelial to the other two somatic cell populations for the sampled stages (Figure S3C). Furthermore, in bovine and pigs, extensive interactions were identified between granulosa and endothelial cells for ECM-mediated laminin/collagen and their receptor pairs (Figures S3A and S3C), and between stromal and endothelial cells (Figures S3B and S3C), while these interactions are sparse in goats. Together, these data strongly support the model for a surge of intercellular signaling at the onset of meiosis, prominently the ECM-mediated cues and the RA-responsive MDK/PTN-NCL axis that functions on creating the niche in early female gonads to support proper development of primordial follicles.

## Discussion

While mammalian gonadal development has been extensively characterized in mice and humans, analogous processes in livestock remain poorly defined. This study provides the first single-cell transcriptomic atlas of fetal bovine ovarian development and integrates these data with goat and pig gonads to uncover conserved and species-specific regulatory programs governing early female gametogenesis in livestock.

In addition to the pluripotent markers such as *POU5F1* or *NANOG*, our analysis revealed a conserved network of germ line regulators, such as *PRDM1*, *STAT3*, and *TFAP2C* across all species, underscoring their essential roles in PGC development and identity. The consistent expression of *CD9* in early PGCs across livestock identifies a practical surface marker for isolating germline populations in non-model organisms for downstream research and applications. Beyond classical markers, we identified additional transcriptional regulators, such as *YY1*, *FOXO1*, *MYC*, *ATF4*, *RAD21*, and *CHD1* across all three species, suggesting broader transcriptional circuitry coupling stress response and mitotic cell-cycle control to germ cell specification and differentiation.

A particularly exciting finding was the high expression of a suite of epigenetic regulators, such as *KAT7*, *MLL10*, *HDAC2*, *MTF2*, *CTCF*, and *HITF*, in germ cells across livestock species. As PGCs undergo extensive epigenomic reprogramming, such as DNA demethylation, imprinting, and X chromosome inactivation to reset the developmental clock,^74^^,^^75^ the conserved expression of these genes strongly implies a critical and previously underappreciated role for their regulatory network in livestock germ cell development. Species-specific features further refine this picture. For example, *SMARCA5,* an SWI/SNF chromatin remodeler essential for meiotic progression in mice,^60^^,^^61^ expressed exclusively in bovine and porcine germ cells, consistent with their progression into meiosis during their sampled stages, whereas goat gonads remained pre-meiotic. These findings highlight both the conservation and evolutionary divergence of meiotic initiation mechanisms across mammals.

Our analysis of the somatic compartment also revealed a core of conserved regulators. In addition to *WT1*, TFs, such as *EMX2*, *SMAD1*, *LEF1*, and *TCF3* are highly expressed in granulosa cells across livestock species, reaffirming the pivotal roles of BMP and WNT/β-catenin signaling in granulosa cell proliferation, differentiation, and primordial follicle activation,^76^^,^^77^^,^^78^ as well as the potential importance of *EMX2*, a key regulator of mouse gonadal and urogenital morphogenesis,^62^^,^^63^ in livestock ovarian development. Interestingly, bovine granulosa cells displayed specific enrichment of *PBX3* and *SOX5*, factors previously linked to primate granulosa cell function and vertebrate sex determination,^27^^,^^65^ suggesting potential ruminant-specific adaptations in granulosa differentiation and follicle assembly.

A defining feature of this study is the identification of the robust MDK/PTN-NCL signaling axis from somatic cells to germ cells in bovine and pigs, which was conspicuously absent in goats. As a pleiotropic heparin-binding growth factor, MDK and its homolog PTN are known to be crucial in embryogenesis, tissue repair, and cancer progression through receptors such as ALK and LRP1. Previous studies have highlighted the importance of MDK/PTN signaling in a variety of biological processes. For example, Jiang^79^ demonstrated that MDK promotes angiogenesis and tumor growth by activating ALK and LRP1 in cancer cells. In reproduction, MDK has been implicated in supporting primordial follicle assembly in mice.^80^ As MDK is a known retinoic acid-responsive factor, the timing of this signaling correlates with the onset of meiosis, suggesting that *MDK/PTN-NCL* interactions form part of a conserved meiotic microenvironment that orchestrates germ-somatic communication and primordial follicle assembly, with its expression known to be regulated by retinoic acid, linking the timing of MDK signaling to the onset of meiosis.^81^ These findings suggest that MDK/PTN-NCL interactions are part of a conserved meiotic microenvironment that orchestrates germ-somatic communication and primordial follicle assembly. Moreover, *MDK* is overexpressed in various human malignancies, including ovarian cancer, and regulates cancer cell growth, survival, migration, and angiogenesis.^82^ The absence of this pathway in goat samples, which have yet to enter meiosis at comparable developmental stages, reinforces its likely role in coordinating meiotic initiation and gonadal microenvironmental remodeling. This spatial-temporal activation pattern, coupled with the extensive ECM-mediated signaling in bovine and porcine gonads, points to a coordinated surge in intercellular crosstalk at the onset of meiosis, which may be a crucial event for constructing proper cellular architecture and providing necessary signals to support the formation of follicle pool in gonads. This is the first identification of the *MDK/PTN-NCL* axis as a meiotic niche signal in mammals.

Our findings support a working model in which the comparatively delayed meiotic initiation observed in the fetal goat ovary arises from coordinated temporal regulation of germ cell-intrinsic and somatic niche-derived signals. In mammals, RA signaling licenses meiotic entry through activation of *STRA8* and associated regulators.^83^ We propose that species-specific differences in RA dynamics or tissue responsiveness shift the window of meiotic competence in goats. Critically, meiotic entry is not solely germ cell-autonomous but depends on a permissive somatic microenvironment. Delayed maturation of pre-granulosa cells and gradual establishment of WNT/BMP-mediated paracrine signaling may transiently constrain germ cell sensitivity to RA, thereby postponing meiotic onset.^84^ Within this framework, *FOXL2* upregulation marks the progressive stabilization of granulosa cell identity and ovarian fate,^85^ reinforcing somatic support necessary for follicle assembly rather than directly triggering meiosis. Concurrently, MDK signaling may modulate germ cell survival and extracellular matrix remodeling during cyst breakdown,^81^ integrating survival and differentiation cues. Together, these processes form a temporally coordinated regulatory network that delays meiotic entry while optimizing primordial follicle establishment, ultimately shaping the slower developmental tempo of the goat ovary.

In conclusion, our comparative single-cell analyses delineate both universal and species-specific gene expression programs, transcriptional regulators, and cell-cell communication events underlying early ovarian development in livestock. This work establishes a valuable resource for the reproductive biology community and offers a framework for future functional and translational studies, ranging from fertility optimization and genetic improvement in breeding programs to advances in *in vitro* gametogenesis.

### Limitations of the study

Despite providing a comprehensive single-cell atlas of bovine fetal ovarian development and cross-species comparisons, several limitations should be acknowledged.

First, this study is primarily based on transcriptomic data, and the inferred cellular states, developmental trajectories, and regulatory networks lack direct functional validation. Future studies integrating genetic or *in vitro* functional assays will be necessary to confirm the roles of identified key regulators and signaling pathways. Second, although cross-species integration was performed using orthologous genes, potential biases arising from differences in genome annotation, sequencing depth, and sample collection stages across species may influence the comparative analyses. Therefore, some species-specific differences observed in this study should be interpreted with caution. Third, the developmental stages analyzed in this study, while covering key transitions from sex determination to early folliculogenesis, may not fully capture the complete dynamics of germ cell maturation, particularly later stages of meiosis and follicle development. Finally, cell-cell communication analysis was inferred computationally based on ligand-receptor expression and does not directly measure physical interactions or signaling activity. Experimental validation will be required to confirm the predicted signaling pathways, including RA-responsive and MDK/PTN-NCL signaling.

## Resource availability

### Lead contact

Further information and requests for resources should be directed to the lead contact, Young Tang (youngtang@nwafu.edu.cn).

### Materials availability

This study did not generate new unique reagents.

### Data and code availability

Data: Single-cell RNA-seq data have been deposited in CNCB under accession OMIX012725 and are publicly available.
Code: Code is available from the corresponding author upon reasonable request.
Additional Information: Any additional information required is available from the lead contact.

## Acknowledgments

This work was supported by the Biological Breeding-National Science and Technology Major Project (2023ZD0407504) and the 10.13039/501100005236Chinese Universities Scientific Fund (245-2023-F2010123001 and 245-2025-Z1090224009). Additional support was provided by the National Science Foundation (grant #2213824).

## Author contributions

S.H., conceptualization, methodology, software, validation, investigation, writing – original draft, and review and editing; Q.Z., software, investigation, formal analysis, data curation, and visualization; Y.F., software, resources, investigation, and writing – review and editing; J.Z., validation and formal analysis; Y.S., investigation; S.X., data curation and formal analysis; Z.Y., investigation and visualization; Y.Z., resources; N.L., investigation; W.W., visualization; L.Y., resources; H.X., investigation; Y.W., supervision, project administration, and writing – review and editing; E.D., supervision, funding acquisition, and writing – review and editing; Y.T., supervision, conceptualization, formal analysis, funding acquisition, and writing – original draft; all authors have read and agreed to the published version of the manuscript.

## Declaration of interests

The authors declare no competing interests.

## STAR★Methods

### Key resources table

**Table:** 

REAGENT or RESOURCE	SOURCE	IDENTIFIER
**Biological samples**		

Bovine female embryonic tissues (E46, E73, E83, E92and E112)	local slaughterhouse	N/A

**Critical commercial assays**		

Single Cell 3' RNA-Seq Kit (v2.1)	MobiDrop	cat. no. S050200201
microfluidic chip of Chip A Single Cell Kit	MobiDrop	cat. no. S050100201

**Deposited data**		

Single-cell RNA Sequencing - raw data	This paper	CNCB: OMIX012725

**Software and algorithms**		

Cell Ranger(3.1)	10x Genomics	https://support.10xgenomics.com/single-cell-gene-expression/software/overview/welcome
Seurat package (v5.2.0)	Hao & Hao et al., 2021	https://satijalab.org/seurat/
pySCENIC (v0.11.2)	Van de Sande et al.^86^	https://pyscenic.readthedocs.io/en/latest/scenic.readthedocs.io/en/latest/
Monocle3	Trapnell Lab	https://cole-trapnell-lab.github.io/monocle3/
CellChat package (v1.6.1)	Jin et al., 2021;	https://github.com/sqjin/CellChat
org.Hs.eg.db (v3.13.0)	Wu et al., 2021	https://bioconductor.org/packages/release/data/annotation/html/org.Hs.eg.db.html
CellPhoneDB (v2.14.0)	Efremova et al., 2020	https://www.cellphonedb.org/

### Experimental model and study participant details

#### Animal models

Bovine fetal gonads were collected from female embryos at stages E38, E46, E73, E83, E92, and E112. Single-cell RNA sequencing data for fetal bovine (E38, E46, E73, E83, E92, E112) are available in the China National Center for Bioinformation (CNCB) database under accession number CNCB: OMIX012725.

Single-cell RNA sequencing data for pig and goat used in this study are publicly available in the Zenodo database (hosted by CERN) under accession number:zenodo.6918355.

Single-cell RNA sequencing data for bovine (E50) used in this study are publicly available in GEO (hosted by NCBI) under accession number: GSE162952.

#### Ethics statement

All animal experimental procedures were performed in accordance with the National Research Council's Guide for the Care and Use of Laboratory Animals and were approved by the Institutional Animal Care and Use Committee (IACUC) of Northwest A&F University (protocol code: IACUC2024-1204).

### Method details

#### Fetal bovine gonad collection

Estrus signs of donor cows raised under natural conditions were observed daily to confirm pregnancy. Artificial insemination was performed once at the onset of detected estrus. Uteri were collected from cows slaughtered in a local slaughterhouse and transferred to a sterile laboratory for disinfection. Embryos were then dissected, and gonads were isolated. Morphologically intact fetal gonads with adequate cell viability were collected from both female embryos at stages E38, E46, E73, E83, E92and E112.

**Table:** Sample information for single-cell RNA sequencing of bovine fetal gonads.

Sample ID	Breed	Sex	Gestational Age (days)	body weight(g)	Crown-Rump Length(cm)
F1	Simmental	Famale	112	638.26	24.5
F2	Simmental	Famale	92	339.24	18.1
F3	Simmental	Famale	83	170.97	15.4
F4	Simmental	Famale	73	91,89	12.6
F6	Simmental	Famale	46	9.72	2.9
F7	Simmental	Famale	38	3.47	1.7

#### Single-cell suspension preparation of fetal gonads

To collect Primordial Germ Cells (PGCs) and gonadal somatic cells, gonads were dissected under a stereomicroscope and transferred to a 1.5 mL tube containing a digestion mix of 1 mg/mL Collagenase IV, 1 mg/mL Hyaluronidase, 0.25% Trypsin, and 1 mg/mL DNase I, followed by incubation at 37°C for 30 minutes. After digestion, serum was added to neutralize the enzymes. The cell suspension was pipetted repeatedly, filtered through a 40 μm cell strainer, and resuspended in 0.1% serum/PBS to prepare single-cell suspensions for 10x Genomics single-cell RNA-seq.

#### Single-cell RNA sequencing

The viability of the single-cell suspension exceeded 80%, and the cell concentration was adjusted to 700-1200 cells/μL. The cells of each sample were then processed with the MobiCube® High Throughput Single Cell 3' RNA-Seq Kit (v2.1) as per manufacturer' s instruction in Novogene Bioinformatics Technology Co., Ltd (Tianjing,China). The cells/nuclei were loaded into microfluidic chip of Chip A Single Cell Kit (MobiDrop, cat. no. S050100201) to generate droplets with MobiNova-100 (MobiDrop, cat. no.A1A40001). Each cell/nuclus was involved into a droplet which contained a gel bead linked with up to millions oligos (cell unique barcode). After encapsulation, droplets suffer light cut by MobiNovaSP-100 (MobiDrop, cat. no.A2A40001) while oligos diffuse into reaction mix. The mRNAs were captured by gel beads containing oligo(dT) in droplets. Following reverse transcription, cDNAs with barcodes were amplified, and a library was constructed using the High Throughput Single Cell 3’RNA-Seq Kit v2.0 (MobiDrop (Zhejiang) Co., Ltd., cat. no. S050200201) and the 3' Single Index Kit (MobiDrop (Zhejiang) Co., Ltd., cat. no. S050300201). Libraries were sequenced by the Illumina NovaSeq sequencer with 150-bp paired-end reads in Novogene Bioinformatics Technology Co. Ltd (Tianjing,China).

#### RNA-seq library preparation and sequencing

Total RNA was isolated using the miRNeasy Mini Kit (Qiagen, 217004), with integrity verified on an Agilent Bioanalyzer 2100 system. The RNA was then fragmented in First-Strand Synthesis Reaction Buffer (5X) using divalent cations at elevated temperature. First-strand cDNA was synthesized with random hexamer primers and M-MuLV Reverse Transcriptase (RNase H-), followed by second-strand synthesis using DNA Polymerase I and RNase H. Subsequent library fragments were purified and PCR-amplified with Phusion High-Fidelity DNA polymerase, universal PCR primers, and a unique index primer. The final PCR products were purified using the AMPure XP system, and library quality was assessed on the Bioanalyzer. Sequencing was performed by Novogene on an Illumina NovaSeq 6000 platform, generating approximately 50 million paired-end 150 bp reads per sample.

#### Single-cell RNA sequencing data processing

Raw sequencing data were processed using Cell Ranger (v3.1, 10x Genomics). Sequencing reads were aligned to the respective reference genomes. The reference genomes for cattle (ARS1), pig (Sscrofa11.1), and goat (ARS1) were built according to Cell Ranger instructions, with genome sequences and annotations obtained from Ensemble (release 98). The filtered feature-barcode matrices generated by Cell Ranger were used for subsequent analysis.

#### Cell identification

Based on differential expression analysis of this dataset and referenced published mammalian ovarian scRNA-seq studies^22^

#### Cell clustering and annotation

Preliminary analysis of the single-cell data was performed using the Seurat package (v5.2.0). Low-quality cells expressing fewer than 300 genes and genes detected in fewer than 3 cells were filtered out. Cells with a mitochondrial gene count percentage exceeding 10% were also removed from the dataset. Data were normalized based on the top 2000 highly variable genes. Batch effects were corrected with Harmony. The Harmony-corrected data were subsequently used for dimensionality reduction, clustering, and manual cell type annotation based on the identification of cluster-specific marker genes in accordance following Seurat guidelines.

#### Differential expression gene (DEG) analysis and Gene Ontology (GO) enrichment

For each cell type, differential expression genes (DEGs) were identified using the Wilcoxon rank-sum test. Significance for both differential expression analysis and GO enrichment analysis was corrected for multiple testing using the Benjamini-Hochberg method, with an adjusted p-value (adj. p-val) < 0.05 considered statistically significant. For significant DEGs identified in specific cell types, Gene Ontology (GO) and KEGG pathway enrichment analyses were performed using the clusterProfiler package (v4.2.1) to identify enriched biological processes and pathways.^87^

#### Transcriptional regulatory network analysis (SCENIC)

Gene regulatory network analysis was conducted using the Python package pySCENIC (v0.11.2). The analysis comprised three main steps: 1) Identification of co-expression modules between transcription factors (TFs) and potential target genes based on correlation; 2) Formulation and refinement of these modules, followed by motif enrichment analysis to define regulons (a TF and its direct target genes); 3) Evaluation of regulon activity in individual cells. Transcription factors with high weights and confidence were retained for downstream analyses, with correlation thresholds adjusted flexibly based on the number of pairs.^86^

#### Pseudotime and cell trajectory inference

Pseudotime analysis and cell differentiation trajectory inference were performed using the Monocle3 package.^88^ Cells were ordered along pseudotime, and UMAP was applied for dimensionality reduction. Following quality control and preliminary cell subtype annotation, which revealed a basic developmental hierarchy, cells with a progenitor state were manually specified as the trajectory starting point based on the expression of canonical marker genes and biological knowledge. The graph_test function in Monocle3 was used to identify genes correlated with the reconstructed developmental trajectory, allowing for the assessment of dynamic gene expression changes along the path.

#### Cell-cell communication analysis

Cell-cell communication analysis was performed using the CellChat package (v1.6.1).^66^ Based on a comprehensive database of known ligand-receptor interactions and integrated with cell type annotations, signaling relationships between different cell types were systematically inferred. By calculating communication probabilities and signal strength, intercellular interaction differences during early PGC development across species were explored. Then, the overall interaction network and specific signaling pathways were visualized.

### Quantification and statistical analysis

All statistical analyses were performed using R (version 4.2.0) unless otherwise specified. For single-cell RNA-seq data, differential gene expression analysis was conducted using the Wilcoxon rank-sum test implemented in the Seurat package. P values were adjusted for multiple testing using the Benjamini–Hochberg method, and genes with an adjusted P value (FDR) < 0.05 were considered statistically significant.

For Gene Ontology (GO) and pathway enrichment analyses, adjusted P values (false discovery rate, FDR) were calculated to control for multiple comparisons. Only significantly enriched terms with FDR < 0.05 were retained for downstream interpretation.

In cell–cell communication analysis using CellChat, communication probabilities were inferred based on the expression of ligand–receptor pairs, and statistical significance was determined using permutation tests implemented in the package.

## Supplemental information

Document S1. Figures S1–S3

Table S1. Summary of cell-type-specific marker genes identified across all bovine gonadal cell clusters

Table S2. Integrated cluster marker genes across bovine, goat, and pig gonadal single-cell datasets

Table S3. Differentially expressed genes in germ cells across developmental stages

Table S4. Differentially expressed genes in granulosa cells during ovarian development

Table S5. Shared and unique gene sets identified in germ cells based on Venn analysis

Table S6. Gene Ontology (GO) enrichment analysis of transcription factors in germ cells

Table S7. Transcription factor analysis in germ cells

Table S8. Shared and unique gene sets in granulosa cells identified by Venn analysis

Table S9. Gene Ontology (GO) enrichment analysis of transcription factors in granulosa cells

Table S10. Transcription factor analysis in granulosa cells

## References

[1] Mackay S. (2000). Gonadal development in mammals at the cellular and molecular levels. Int. Rev. Cytol..

[2] Clinton M. (1998). Sex determination and gonadal development: a bird's eye view. J. Exp. Zool..

[3] Gunes S.O., Metin Mahmutoglu A., Agarwal A. (2016). Genetic and epigenetic effects in sex determination. Birth Defects Res. C Embryo Today..

[4] Chassot A.A., Ranc F., Gregoire E.P., Roepers-Gajadien H.L., Taketo M.M., Camerino G., de Rooij D.G., Schedl A., Chaboissier M.C. (2008). Activation of beta-catenin signaling by Rspo1 controls differentiation of the mammalian ovary. Hum. Mol. Genet..

[5] Kim Y., Kobayashi A., Sekido R., DiNapoli L., Brennan J., Chaboissier M.C., Poulat F., Behringer R.R., Lovell-Badge R., Capel B. (2006). Fgf9 and Wnt4 act as antagonistic signals to regulate mammalian sex determination. PLoS Biol..

[6] Maatouk D.M., DiNapoli L., Alvers A., Parker K.L., Taketo M.M., Capel B. (2008). Stabilization of beta-catenin in XY gonads causes male-to-female sex-reversal. Hum. Mol. Genet..

[7] Tomizuka K., Horikoshi K., Kitada R., Sugawara Y., Iba Y., Kojima A., Yoshitome A., Yamawaki K., Amagai M., Inoue A. (2008). R-spondin1 plays an essential role in ovarian development through positively regulating Wnt-4 signaling. Hum. Mol. Genet..

[8] Vainio S., Heikkilä M., Kispert A., Chin N., McMahon A.P. (1999). Female development in mammals is regulated by Wnt-4 signalling. Nature.

[9] Yao H.H.C., Matzuk M.M., Jorgez C.J., Menke D.B., Page D.C., Swain A., Capel B. (2004). Follistatin operates downstream of Wnt4 in mammalian ovary organogenesis. Dev. Dyn..

[10] Garcia-Ortiz J.E., Pelosi E., Omari S., Nedorezov T., Piao Y., Karmazin J., Uda M., Cao A., Cole S.W., Forabosco A. (2009). Foxl2 functions in sex determination and histogenesis throughout mouse ovary development. BMC Dev. Biol..

[11] Ottolenghi C., Pelosi E., Tran J., Colombino M., Douglass E., Nedorezov T., Cao A., Forabosco A., Schlessinger D. (2007). Loss of Wnt4 and Foxl2 leads to female-to-male sex reversal extending to germ cells. Hum. Mol. Genet..

[12] Koopman P., Gubbay J., Vivian N., Goodfellow P., Lovell-Badge R. (1991). Male development of chromosomally female mice transgenic for Sry. Nature.

[13] Larney C., Bailey T.L., Koopman P. (2014). Switching on sex: transcriptional regulation of the testis-determining gene Sry. Development.

[14] Munger S.C., Natarajan A., Looger L.L., Ohler U., Capel B. (2013). Fine time course expression analysis identifies cascades of activation and repression and maps a putative regulator of mammalian sex determination. PLoS Genet..

[15] Nef S., Stévant I., Greenfield A. (2019). Characterizing the bipotential mammalian gonad. Curr. Top. Dev. Biol..

[16] Ohinata Y., Payer B., O'Carroll D., Ancelin K., Ono Y., Sano M., Barton S.C., Obukhanych T., Nussenzweig M., Tarakhovsky A. (2005). Blimp1 is a critical determinant of the germ cell lineage in mice. Nature.

[17] Weber S., Eckert D., Nettersheim D., Gillis A.J.M., Schäfer S., Kuckenberg P., Ehlermann J., Werling U., Biermann K., Looijenga L.H.J., Schorle H. (2010). Critical function of AP-2 gamma/TCFAP2C in mouse embryonic germ cell maintenance. Biol. Reprod..

[18] Alves-Lopes J.P., Wong F.C.K., Tang W.W.C., Gruhn W.H., Ramakrishna N.B., Jowett G.M., Jahnukainen K., Surani M.A. (2023). Specification of human germ cell fate with enhanced progression capability supported by hindgut organoids. Cell Rep..

[19] Tang W.W.C., Dietmann S., Irie N., Leitch H.G., Floros V.I., Bradshaw C.R., Hackett J.A., Chinnery P.F., Surani M.A. (2015). A Unique Gene Regulatory Network Resets the Human Germline Epigenome for Development. Cell.

[20] Irie N., Weinberger L., Tang W.W.C., Kobayashi T., Viukov S., Manor Y.S., Dietmann S., Hanna J.H., Surani M.A. (2015). SOX17 is a critical specifier of human primordial germ cell fate. Cell.

[21] Chan M.E., Bhamidipati P.S., Goldsby H.J., Hintze A., Hofmann H.A., Young R.L. (2020). Comparative transcriptomics reveal distinct patterns of gene expression conservation through vertebrate embryogenesis. bioRxiv.

[22] Chen M., Long X., Chen M., Hao F., Kang J., Wang N., Wang Y., Wang M., Gao Y., Zhou M. (2022). Integration of single-cell transcriptome and chromatin accessibility of early gonads development among goats, pigs, macaques, and humans. Cell Rep..

[23] Schmidt D., Ovitt C.E., Anlag K., Fehsenfeld S., Gredsted L., Treier A.C., Treier M. (2004). The murine winged-helix transcription factor Foxl2 is required for granulosa cell differentiation and ovary maintenance. Development.

[24] Nagahama Y., Chakraborty T., Paul-Prasanth B., Ohta K., Nakamura M. (2021). Sex determination, gonadal sex differentiation, and plasticity in vertebrate species. Physiol. Rev..

[25] [dataset] (2021). Single-cell RNA-sequencing of bovine fetal gonads using 10x Genomics. Géo.

[26] Mayère C., Neirijnck Y., Sararols P., Rands C.M., Stévant I., Kühne F., Chassot A.A., Chaboissier M.C., Dermitzakis E.T., Nef S. (2021). Single-cell transcriptomics reveal temporal dynamics of critical regulators of germ cell fate during mouse sex determination. FASEB J..

[27] Garcia-Alonso L., Lorenzi V., Mazzeo C.I., Alves-Lopes J.P., Roberts K., Sancho-Serra C., Engelbert J., Marečková M., Gruhn W.H., Botting R.A. (2022). Single-cell roadmap of human gonadal development. Nature.

[28] Ndiaye K., Fayad T., Silversides D.W., Sirois J., Lussier J.G. (2005). Identification of downregulated messenger RNAs in bovine granulosa cells of dominant follicles following stimulation with human chorionic gonadotropin. Biol. Reprod..

[29] Warma A., Ndiaye K. (2020). Functional effects of Tribbles homolog 2 in bovine ovarian granulosa cellsdagger. Biol. Reprod..

[30] Kulus J., Kranc W., Kulus M., Bukowska D., Piotrowska-Kempisty H., Mozdziak P., Kempisty B., Antosik P. (2023). New Gene Markers of Exosomal Regulation Are Involved in Porcine Granulosa Cell Adhesion, Migration, and Proliferation. Int. J. Mol. Sci..

[31] Nicol B., Grimm S.A., Chalmel F., Lecluze E., Pannetier M., Pailhoux E., Dupin-De-Beyssat E., Guiguen Y., Capel B., Yao H.H.C. (2019). RUNX1 maintains the identity of the fetal ovary through an interplay with FOXL2. Nat. Commun..

[32] Powner D., Kopp P.M., Monkley S.J., Critchley D.R., Berditchevski F. (2011). Tetraspanin CD9 in cell migration. Biochem. Soc. Trans..

[33] Sereesongsaeng N., Burrows J.F., Scott C.J., Brix K., Burden R.E. (2023). Cathepsin V regulates cell cycle progression and histone stability in the nucleus of breast cancer cells. Front. Pharmacol..

[34] Lee H., Cho S., Kim M.J., Park Y.J., Cho E., Jo Y.S., Kim Y.S., Lee J.Y., Thoudam T., Woo S.H. (2023). ApoE4-dependent lysosomal cholesterol accumulation impairs mitochondrial homeostasis and oxidative phosphorylation in human astrocytes. Cell Rep..

[35] Wang Q., Liu J., Yang M., Zhou J., Li Y., Zheng J., Jia H., Yue S., Le Y., Su Y. (2025). Targeting AKR1B1 inhibits metabolic reprogramming to reverse systemic therapy resistance in hepatocellular carcinoma. Signal Transduct. Targeted Ther..

[36] Bai F.R., Wu Q.Q., Wu Y.J., Hu Y.Q., Jiang Z.X., Lv H., Qian W.Z., Cai C., Wu J.W. (2022). Germline FOXJ2 overexpression causes male infertility via aberrant autophagy activation by LAMP2A upregulation. Cell Death Dis..

[37] Boulanger L., Pannetier M., Gall L., Allais-Bonnet A., Elzaiat M., Le Bourhis D., Daniel N., Richard C., Cotinot C., Ghyselinck N.B., Pailhoux E. (2014). FOXL2 is a female sex-determining gene in the goat. Curr. Biol..

[38] Pailhoux E., Vigier B., Vaiman D., Servel N., Chaffaux S., Cribiu E.P., Cotinot C. (2002). Ontogenesis of female-to-male sex-reversal in XX polled goats. Dev. Dyn..

[39] Schneider J., Mitschke J., Bhat M., Vogele D., Schilling O., Reinheckel T., Heß L. (2024). Cathepsin D inhibition during neuronal differentiation selectively affects individual proteins instead of overall protein turnover. Biochimie.

[40] Thedieck C., Kuczyk M., Klingel K., Steiert I., Müller C.A., Klein G. (2005). Expression of Ksp-cadherin during kidney development and in renal cell carcinoma. Br. J. Cancer.

[41] de Cristofaro T., Di Palma T., Fichera I., Lucci V., Parrillo L., De Felice M., Zannini M. (2012). An essential role for Pax8 in the transcriptional regulation of cadherin-16 in thyroid cells. Mol. Endocrinol..

[42] Li X., Wang L., Liang X.Y., Zhang H.W., Shi J.G., Guo J., Zou D.F., Chen J., He S.L., Xie Y.P. (2025). Variants in CSMD2 and CSMD3, genes involved in synaptogenesis, are associated with epilepsies. Epilepsia.

[43] Matsunuma R., Chan D.W., Kim B.J., Singh P., Han A., Saltzman A.B., Cheng C., Lei J.T., Wang J., Roberto da Silva L. (2018). DPYSL3 modulates mitosis, migration, and epithelial-to-mesenchymal transition in claudin-low breast cancer. Proc. Natl. Acad. Sci. USA.

[44] Shang N., Lee J.T.Y., Huang T., Wang C., Lee T.L., Mok S.C., Zhao H., Chan W.Y. (2020). Disabled-2: a positive regulator of the early differentiation of myoblasts. Cell Tissue Res..

[45] François M., Caprini A., Hosking B., Orsenigo F., Wilhelm D., Browne C., Paavonen K., Karnezis T., Shayan R., Downes M. (2008). Sox18 induces development of the lymphatic vasculature in mice. Nature.

[46] Yan H., Kermouni A., Abdel-Hafez M., Lau D.C.W. (2003). Role of cyclooxygenases COX-1 and COX-2 in modulating adipogenesis in 3T3-L1 cells. J. Lipid Res..

[47] Chuang S.C., Chou Y.S., Lin Y.S., Chang J.K., Chen C.H., Ho M.L. (2025). Cyclooxygenase-2 negatively regulates osteogenic differentiation in murine bone marrow mesenchymal stem cells via the FOXO3a/p27kip1 pathway. Bone Joint Res..

[48] Wang C.Y., Tang M.C., Chang W.C., Furushima K., Jang C.W., Behringer R.R., Chen C.M. (2016). PiggyBac Transposon-Mediated Mutagenesis in Rats Reveals a Crucial Role of Bbx in Growth and Male Fertility. Biol. Reprod..

[49] Zhang R., Tu Y.X., Ye D., Gu Z., Chen Z.X., Sun Y. (2022). A Germline-Specific Regulator of Mitochondrial Fusion is Required for Maintenance and Differentiation of Germline Stem and Progenitor Cells. Adv. Sci..

[50] Zhang Y., Wang Y., Feng X., Zhang S., Xu X., Li L., Niu S., Bo Y., Wang C., Li Z. (2021). Oocyte-derived microvilli control female fertility by optimizing ovarian follicle selection in mice. Nat. Commun..

[51] Pastor W.A., Liu W., Chen D., Ho J., Kim R., Hunt T.J., Lukianchikov A., Liu X., Polo J.M., Jacobsen S.E., Clark A.T. (2018). TFAP2C regulates transcription in human naive pluripotency by opening enhancers. Nat. Cell Biol..

[52] Kim J., Driscoll C.S., Li L., Wilson C.A., Xie W., Knott J.G. (2025). Deciphering the role of cis-regulatory elements and TFAP2C in the activation of zygotic Sox2 expression in mouse preimplantation embryos. Development.

[53] Cuitiño M.C., Pécot T., Sun D., Kladney R., Okano-Uchida T., Shinde N., Saeed R., Perez-Castro A.J., Webb A., Liu T. (2019). Two Distinct E2F Transcriptional Modules Drive Cell Cycles and Differentiation. Cell Rep..

[54] Christensen J., Cloos P., Toftegaard U., Klinkenberg D., Bracken A.P., Trinh E., Heeran M., Di Stefano L., Helin K. (2005). Characterization of E2F8, a novel E2F-like cell-cycle regulated repressor of E2F-activated transcription. Nucleic Acids Res..

[55] Musa J., Aynaud M.M., Mirabeau O., Delattre O., Grünewald T.G. (2017). MYBL2 (B-Myb): a central regulator of cell proliferation, cell survival and differentiation involved in tumorigenesis. Cell Death Dis..

[56] Han Q., Guo X., Jia K., Jing J., Dang W., Li Y., Qin X., Li P., Ren Y., Liu W. (2020). Effects of FOXO1 on the proliferation and cell cycle-apoptosis- and steroidogenesis-related genes expression in sheep granulosa cells. Anim. Reprod. Sci..

[57] Torrence M.E., MacArthur M.R., Hosios A.M., Valvezan A.J., Asara J.M., Mitchell J.R., Manning B.D. (2021). The mTORC1-mediated activation of ATF4 promotes protein and glutathione synthesis downstream of growth signals. eLife.

[58] Masuoka H.C., Townes T.M. (2002). Targeted disruption of the activating transcription factor 4 gene results in severe fetal anemia in mice. Blood.

[59] Ma P., Schultz R.M. (2016). HDAC1 and HDAC2 in mouse oocytes and preimplantation embryos: Specificity versus compensation. Cell Death Differ..

[60] Kataruka S., Malla A.B., Rainsford S.R., Walters B.W., Heuer R.A., Marshall K.L., Lesch B.J. (2025). SMARCA5 restricts chromatin accessibility to promote male meiosis and fertility in mammals. Proc. Natl. Acad. Sci. USA.

[61] Zhang C., Chen Z., Yin Q., Fu X., Li Y., Stopka T., Skoultchi A.I., Zhang Y. (2020). The chromatin remodeler Snf2h is essential for oocyte meiotic cell cycle progression. Genes Dev..

[62] Miyamoto N., Yoshida M., Kuratani S., Matsuo I., Aizawa S. (1997). Defects of urogenital development in mice lacking Emx2. Development.

[63] Kusaka M., Katoh-Fukui Y., Ogawa H., Miyabayashi K., Baba T., Shima Y., Sugiyama N., Sugimoto Y., Okuno Y., Kodama R. (2010). Abnormal epithelial cell polarity and ectopic epidermal growth factor receptor (EGFR) expression induced in Emx2 KO embryonic gonads. Endocrinology.

[64] Chen M., Cen C., Wang N., Shen Z., Wang M., Liu B., Li J., Cui X., Wang Y., Gao F. (2022). The functions of Wt1 in mouse gonad development and somatic cells differentiationdagger. Biol. Reprod..

[65] Schartl M., Schories S., Wakamatsu Y., Nagao Y., Hashimoto H., Bertin C., Mourot B., Schmidt C., Wilhelm D., Centanin L. (2018). Sox5 is involved in germ-cell regulation and sex determination in medaka following co-option of nested transposable elements. BMC Biol..

[66] Jin S., Plikus M.V., Nie Q. (2025). CellChat for systematic analysis of cell-cell communication from single-cell transcriptomics. Nat. Protoc..

[67] Merkwitz C., Lochhead P., Tsikolia N., Koch D., Sygnecka K., Sakurai M., Spanel-Borowski K., Ricken A.M. (2011). Expression of KIT in the ovary, and the role of somatic precursor cells. Prog. Histochem. Cytochem..

[68] Kurek M., Åkesson E., Yoshihara M., Oliver E., Cui Y., Becker M., Alves-Lopes J.P., Bjarnason R., Romerius P., Sundin M. (2021). Spermatogonia Loss Correlates with LAMA 1 Expression in Human Prepubertal Testes Stored for Fertility Preservation. Cells.

[69] Bu T., Wang L., Wu X., Li L., Mao B., Wong C.K.C., Perrotta A., Silvestrini B., Sun F., Cheng C.Y. (2022). A laminin-based local regulatory network in the testis that supports spermatogenesis. Semin. Cell Dev. Biol..

[70] Mitsiadis T.A., Salmivirta M., Muramatsu T., Muramatsu H., Rauvala H., Lehtonen E., Jalkanen M., Thesleff I. (1995). Expression of the heparin-binding cytokines, midkine (MK) and HB-GAM (pleiotrophin) is associated with epithelial-mesenchymal interactions during fetal development and organogenesis. Development.

[71] Kadomatsu K., Huang R.P., Suganuma T., Murata F., Muramatsu T. (1990). A retinoic acid responsive gene MK found in the teratocarcinoma system is expressed in spatially and temporally controlled manner during mouse embryogenesis. J. Cell Biol..

[72] Jee Y.H., Lebenthal Y., Chaemsaithong P., Yan G., Peran I., Wellstein A., Romero R., Baron J. (2016). Midkine and Pleiotrophin Concentrations in Amniotic Fluid in Healthy and Complicated Pregnancies. PLoS One.

[73] Cadenas J., Pors S.E., Hansen C.P., Olufsen S.M., Subiran C., Bøtkjær J.A., La Cour Poulsen L., Fedder J., Dueholm M., Colmorn L.B. (2023). Midkine characterization in human ovaries: potential new variants in follicles. F. S. Sci..

[74] Gruhn W.H., Tang W.W.C., Dietmann S., Alves-Lopes J.P., Penfold C.A., Wong F.C.K., Ramakrishna N.B., Surani M.A. (2023). Epigenetic resetting in the human germ line entails histone modification remodeling. Sci. Adv..

[75] Ramakrishna N.B., Murison K., Miska E.A., Leitch H.G. (2021). Epigenetic Regulation during Primordial Germ Cell Development and Differentiation. Sex. Dev..

[76] Habara O., Logan C.Y., Kanai-Azuma M., Nusse R., Takase H.M. (2021). WNT signaling in pre-granulosa cells is required for ovarian folliculogenesis and female fertility. Development.

[77] Su Y.Q., Wu X., O'Brien M.J., Pendola F.L., Denegre J.N., Matzuk M.M., Eppig J.J. (2004). Synergistic roles of BMP15 and GDF9 in the development and function of the oocyte-cumulus cell complex in mice: genetic evidence for an oocyte-granulosa cell regulatory loop. Dev. Biol..

[78] Rossi R.O.D.S., Costa J.J.N., Silva A.W.B., Saraiva M.V.A., Van den Hurk R., Silva J.R.V. (2016). The bone morphogenetic protein system and the regulation of ovarian follicle development in mammals. Zygote.

[79] Yıldırım B., Kulak K., Bilir A. (2025). Midkine (MDK) in cancer and drug resistance: from inflammation to therapy. Discov. Oncol..

[80] Luo M., Yang X., Zhou M., Zhang J., Yu B., Lian H., Ye J. (2024). Integrated single-cell and spatial transcriptomics reveal microenvironment disruptions by androgen in mouse ovary. iScience.

[81] Yu X., Xie L., Ge J., Li H., Zhong S., Liu X. (2023). Integrating single-cell RNA-seq and spatial transcriptomics reveals MDK-NCL dependent immunosuppressive environment in endometrial carcinoma. Front. Immunol..

[82] Filippou P.S., Karagiannis G.S., Constantinidou A. (2020). Midkine (MDK) growth factor: a key player in cancer progression and a promising therapeutic target. Oncogene.

[83] Koubova J., Menke D.B., Zhou Q., Capel B., Griswold M.D., Page D.C. (2006). Retinoic acid regulates sex-specific timing of meiotic initiation in mice. Proc. Natl. Acad. Sci. USA.

[84] Shi Y., Guo Y., Zhou J., Cui G., Cheng J.C., Wu Y., Zhao Y.L., Fang L., Han X., Yang Y.G., Sun Y. (2023). A spatiotemporal gene expression and cell atlases of the developing rat ovary. Cell Prolif..

[85] Uhlenhaut N.H., Jakob S., Anlag K., Eisenberger T., Sekido R., Kress J., Treier A.C., Klugmann C., Klasen C., Holter N.I. (2009). Somatic sex reprogramming of adult ovaries to testes by FOXL2 ablation. Cell.

[86] Van de Sande B., Flerin C., Davie K., De Waegeneer M., Hulselmans G., Aibar S., Seurinck R., Saelens W., Cannoodt R., Rouchon Q. (2020). A scalable SCENIC workflow for single-cell gene regulatory network analysis. Nat. Protoc..

[87] Yu G., Wang L.G., Han Y., He Q.Y. (2012). clusterProfiler: an R package for comparing biological themes among gene clusters. OMICS.

[88] Qiu X., Mao Q., Tang Y., Wang L., Chawla R., Pliner H.A., Trapnell C. (2017). Reversed graph embedding resolves complex single-cell trajectories. Nat. Methods.

