# Supplementary material for: Integration of single-cell transcriptome of female early gonadal development in bovine, goats, and pigs
Source: iScience. 2026 May 22;29(6):116074. doi: 10.1016/j.isci.2026.116074 (PMC13224005; doi:10.1016/j.isci.2026.116074)
Supplement: Document S1. Figures S1–S3 [file mmc1.pdf]

iScience, Volume *29*

Supplemental information

Integration of single-cell transcriptome of female early gonadal development in bovine, goats, and pigs

Shiyao Han, Qianhui Zou, Yifei Fang, Junmei Zhang, Yue Su, Shengcan Xie, Zhen Yang, Yiyu Zhao, Ningxiao Li, Wenjing Wan, Linxiu Yue, Heshuangyi Xie, Yulei Wei, Ellie Duan, and Young Tang

Supplemental information

Integration of Single-cell Transcriptome of Female Early Gonadal Development in Bovine, Goats, and Pigs

Shiyao Han, Qianhui Zou, Yifei Fang, Junmei Zhang, Yue Su, Shengcan Xie, Zhen Yang, Yiyu Zhao, Ningxiao Li, Wenjing Wan, Linxiu Yue, Heshuangyi Xie, Yulei Wei, Ellie Duan, Young Tang

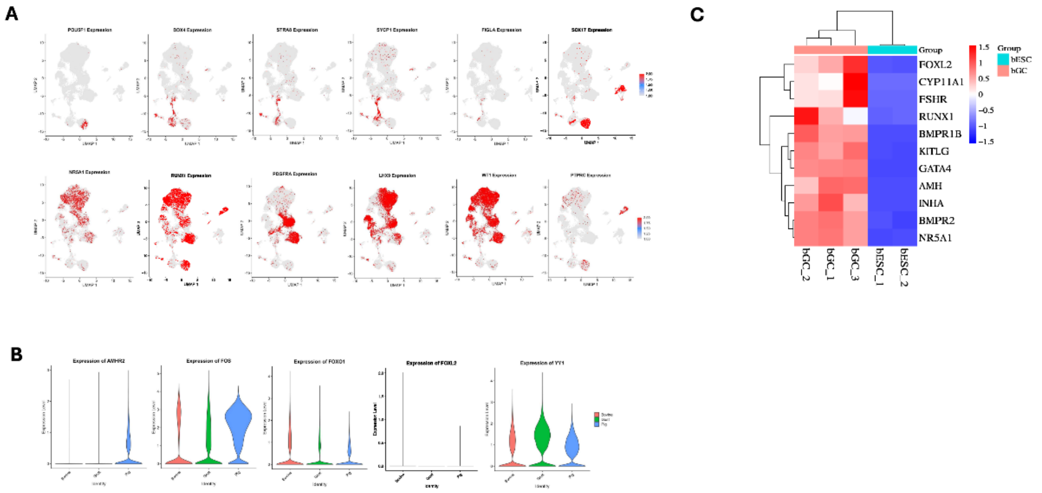

Figure S1: Cellular Landscape and Gene Expression Characteristics of Gonadal Cells.

(A). Uniform Manifold Approximation and Projection (UMAP) visualization of female bovine gonadal germ cells and gonadal somatic cells. Cells are color-coded according to cell type.

(B). The expression levels of some genes for granulosa cells in fetal bovine, goat, and porcine gonads.

(C). Heatmap for gene expression comparison in adult bovine granulosa cells (bGC) and bovine embryonic stem cells (bESC). Values are presented as log2 conversion of normalized counts+1.

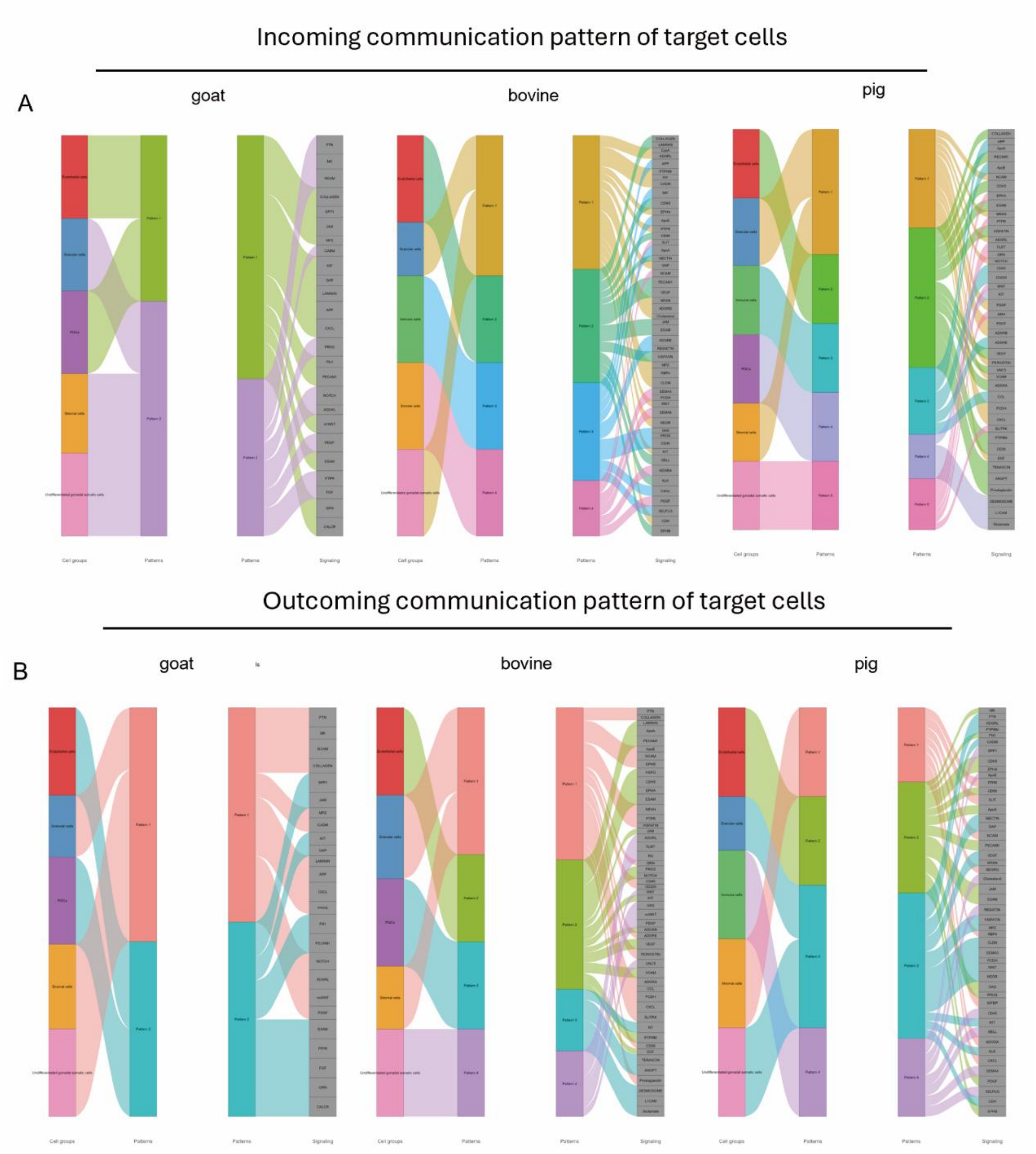

Figure S2: Incoming and Outgoing Signaling Networks in Female Gonadal Cell Populations Across Species

(A). Incoming communication pattern analysis of signaling interactions in female porcine, bovine, and goat gonadal cell populations.

(B). Outgoing communication pattern analysis of signaling interactions in female porcine, bovine, and goat gonadal cell populations.

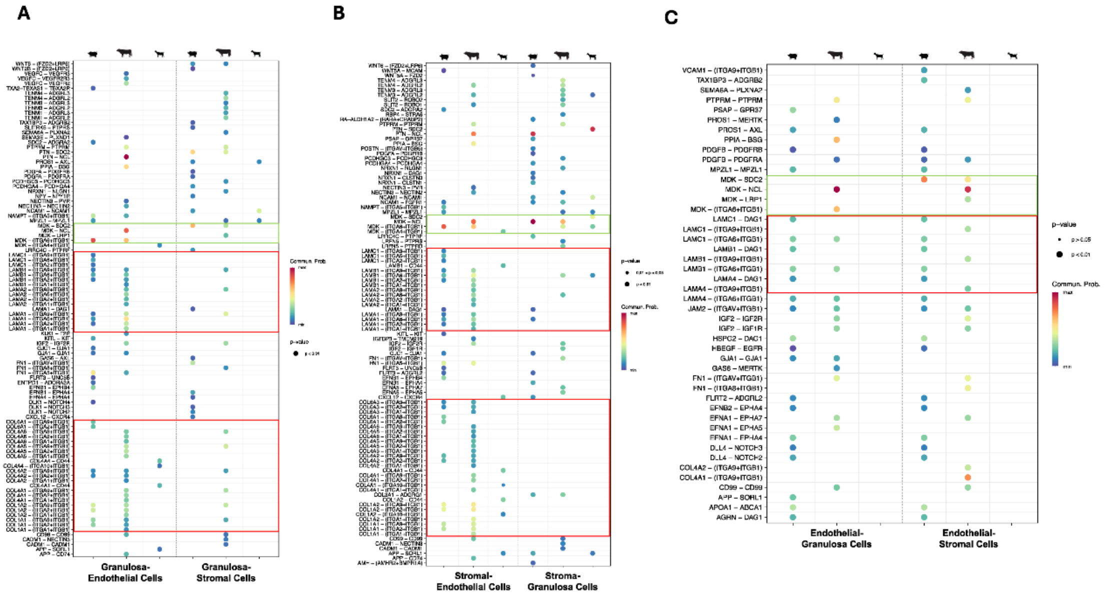

Figure S3: Cell-Type-Specific Ligand–Receptor Interactions Among Gonadal Cell Populations Across Species

(A). Dot plot depicting representative ligand-receptor interactions of gonadal granulosa cells to endothelial and stromal cells in female pigs, bovine, and goats.

(B). Dot plot depicting representative ligand-receptor interactions of gonadal stromal cells to endothelial and granulosa cells in female pigs, bovine, and goats.

(C). Dot plot depicting representative ligand-receptor interactions of endothelial cells to granulosa and stromal cells in female pigs, bovine, and goats.
